# Identification of three novel *GNAO1* variants in a Chinese cohort with *GNAO1* encephalopathy: expanding the clinical and genetic spectrum

**DOI:** 10.1186/s13023-025-03984-x

**Published:** 2025-08-18

**Authors:** Daoqi Mei, Yu Gu, Bingbing Zhang, Shiyue Mei, Xiaona Wang, Yuanning Ma, Jie Deng, Jihong Tang

**Affiliations:** 1 Department of Neurology, Children’s Hospital Affiliated to Suzhou University, No. 92, Zhong Nan Street, Suzhou Industrial Park, Suzhou, 215025 Jiangsu Province China; 2 Department of Pediatrics, Children’s Hospital Affiliated to Zhengzhou University, Henan Children’s Hospital, Zhengzhou Children’s Hospital, 33 Longhu Waihuan East Road, Jinshui District, Zhengzhou, 450018 Henan ProvinceChina China; 3 Henan Provincial Key Laboratory of Children’s Genetics and Metabolic Diseases, Henan Engineering Research Center of Childhood Neurodevelopment, 33 Longhu Waihuan East Road, Jinshui District, Zhengzhou, 450018 Henan Province China; 4 Department of Neurology, Children’s Hospital Affiliated to Zhengzhou University, Henan Children’s Hospital, Zhengzhou Children’s Hospital, 33 Longhu Waihuan East Road, Jinshui District, Zhengzhou, 450018 Henan Province China; 5 Department of Neurology, Beijing Children’s Hospital, No.56 Nanlishi Road, Beijing, 100045 China

**Keywords:** *GNAO1*, Encephalopathy, Epilepsy, Movement disorders, Missense variant

## Abstract

**Objective:**

To summarize the clinical characteristics of a cohort of nine Chinese children with *GNAO1* encephalopathy and analyze their genotypes.

**Methods:**

A retrospective study was conducted on nine children diagnosed with *GNAO1* encephalopathy at the Neurology Department of two children’s hospitals between January 2019 and December 2022. Their clinical manifestations, genetic test results, cranial imaging, electroencephalography and treatment were summarized. Their prognosis was followed up.

**Results:**

All nine patients presented with moderate-to-severe psychomotor developmental delay and dystonia. Six patients exhibited neonatal or infantile-onset epilepsy, manifesting as generalized tonic-clonic seizure, myoclonic seizure, epileptic spasms, and were diagnosed with developmental and epileptic encephalopathy 17 (DEE 17). Two patients presented with choreoathetosis in infancy without epileptic seizure and were diagnosed with the neurodevelopmental disorder with involuntary movements (NEDIM). One patient presented with choreoathetosis at two years of age and developed focal seizures at six years of age, representing an intermediate phenotype. During a follow-up period of 0.8–3.5 years, one child died due to infection. The remaining eight continued to exhibit psychomotor retardation. Pathogenic or likely pathogenic de novo heterozygous missense variants in *GNAO1* were identified in all nine cases. Among these, the variants c.17G > T (p.Ser6Ile), c.119G > C (p.Gly40Ala), and c.748 C > T (p.Leu250Phe) are novel.

**Conclusion:**

In conclusion, we analyzed the clinical characteristics and genetic variants of a cohort of nine Chinese children with *GNAO1* variants and identified three novel *GNAO1* variants. Our study expanded the spectrum of genotypes and phenotypes in *GNOA1*-associated encephalopathy.

**Supplementary Information:**

The online version contains supplementary material available at 10.1186/s13023-025-03984-x.

## Introduction

*GNAO1*-related disorders encompass a broad phenotypic continuum, including hyperkinetic movement disorders and/or epilepsy, often associated with developmental delay and intellectual disability [1]. *GNAO1* encephalopathy is a rare neurodevelopmental disorder that characterized epilepsy and hyperkinetic movement disorders. The clinical phenotypes of patients with *GNAO1* variants are broad and heterogeneous and were first reported in 2013 as developmental and epileptic encephalopathy 17 (DEE17, OMIM 615473), which is an early-infantile onset epileptic encephalopathy [2]. Another phenotype, neurodevelopmental disorder with involuntary movements (NEDIM, OMIM 617493) was then reported as severe hyperkinetic encephalopathy with recurrent dystonic exacerbations and profound developmental delay, with or without seizures [3, 4]. These different phenotypes can be collectively referred as *GNAO1* encephalopathy [5]. Incidence rates in China are currently unknown.

The *GNAO1* encodes the heterotrimeric guanine nucleotide-binding protein (G-protein) α-subunit “o” type (Gαo), one of the most abundant membrane proteins in the central nervous system, which plays an important role in signal transduction through cyclic adenosine monophosphate (cAMP) metabolism in the striatum. Gαo contains guanosine triphosphate (GTP) -binding sites and dissociate from both the G‐protein–coupled receptor and the β‐γ dimer when activated. Gαo activates functionally relevant molecules, such as phospholipase C and calcium channels, in the form of GTP binding, by activating downstream signal transduction to regulate neuronal excitability [6]. To February 2023, 85 patients with *GNAO1* variants have been reported. Most of the variants were missense variants.

Here, we analyzed the clinical characteristics and genetic variants of a cohort of nine Chinese children with *GNAO1* variants and identified three novel *GNAO1* variants. Our study expanded the spectrum of genotypes and phenotypes in *GNOA1*-associated encephalopathy and provided new evidence for clinical diagnosis.

## Materials and methods

This study is a retrospective case series and was approved by the Medical Ethics Committees of two hospitals. Written informed consent for participation in the study, genetic testing, and publication of anonymized clinical data and images was obtained from the legal guardians of all participating children. Nine children diagnosed with *GNAO1* encephalopathy were recruited from January 2019 to December 2022 at the Neurology Department of Beijing Children’s Hospital and Children’s Hospital Affiliated to Suzhou University in this study. The diagnosis of all children was confirmed by clinical features and genetic testing.

Clinical data were collected from the nine children. Laboratory tests included complete blood count, liver and kidney function, thyroid function, blood ammonia, pyruvate, lactate, homocysteine, and blood and urine metabolic screenings. Development was assessed by the Pediatric Neuropsychological Screening Scale (DQ) and the Chinese Wechsler Intelligence Scale for Children (C-WISC). All children underwent echocardiography, abdominal ultrasound, cranial magnetic resonance imaging (MRI) and video electroencephalography (EEG). The efficacy of antiseizure medications (ASMs) was assessed based on seizure frequency reduction from baseline. A reduction of > 50% was considered effective or responsive to treatment, and a reduction of > 90% was considered significantly effective. Seizure freedom was defined as no seizures for at least 6 months at the last follow-up. Follow-up was conducted through outpatient clinic visits and supplemented by telephone interviews where necessary. The final follow-up date for data collection was August 2023.

For genetic testing, 3 ml of anticoagulated blood was collected from each child and their parents. Genomic DNA was extracted and then whole-exome sequencing was performed. The detected target sequences were amplified by polymerase chain reaction and validated by Sanger sequencing using an ABI 3730 sequencer. Validation results were obtained by the sequence analysis software SeqMan to determine the origin of the variants. The reference genome was GRCh37/hg19 and the *GNAO1* gene transcript was NM_020988.3. The pathogenicity of the variants was assessed according to the 2015 American College of Medical Genetics and Genomics (ACMG) guidelines [7]. Gene sequencing was entrusted to Chigene Translational Medical Research Center Co., Ltd., Beijing Kangso Medical Inspection, Co., Ltd., Beijing MyGenostics, Co., Ltd., and Cipher Gene.Data analysis was performed by the Henan Provincial Key Laboratory of Pediatric Genetic and Metabolic Diseases.

## Results

### Clinical manifestations

The cohort comprised six females and three males (*n* = 9), all born at term following uncomplicated perinatal courses. All patients exhibited mental and motor developmental delay. Cases 1–7 presented with seizures and a clinical phenotype consistent with DEE17, while cases 8 and 9 had no seizure and presented marked dystonia and involuntary movements, leading to a diagnosis as NEDIM. Their clinical presentation is detailed in Table 1.

Cases 1-6 developed seizures and hypotonia between two days of age and 2.5 months of age (median age 18 days), while case 7 presented with delayed speech development, intellectual disability (ID), and choreoathetosis at the age of two years and then seizures at the age of six years. Seizure types were as follows: generalized tonic-clonic seizures (cases 1, 3, 4), myoclonic seizures (cases 1, 3, 4 and 5), epileptic spasms (cases 2, 3, 5 and 6) and focal seizures and focal to bilateral tonic-clonic seizures (cases 5–7).

Cases 8 and 9 presented with dystonia and developmental delay at 3–4 months of age, later developing athetosis-chorea during infancy.

**Fig. 1:**
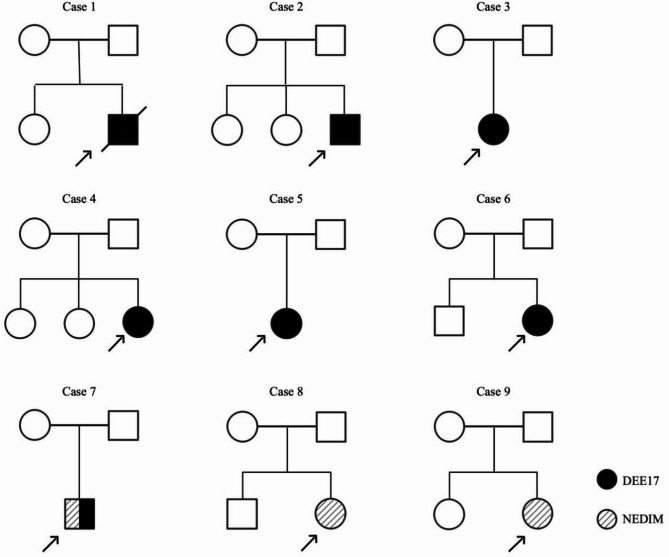
Family pedigrees of nine probands. DEE17, developmental and epileptic encephalopathy 17; NEDIM, neurodevelopmental disorder with involuntary movements

Their parents were non-consanguineous and were all asymptomatic. Siblings of the other six patients were healthy (Fig. 1), except for cases 3, 5 and 7, who were the only child in their families.

**Fig. 2:**
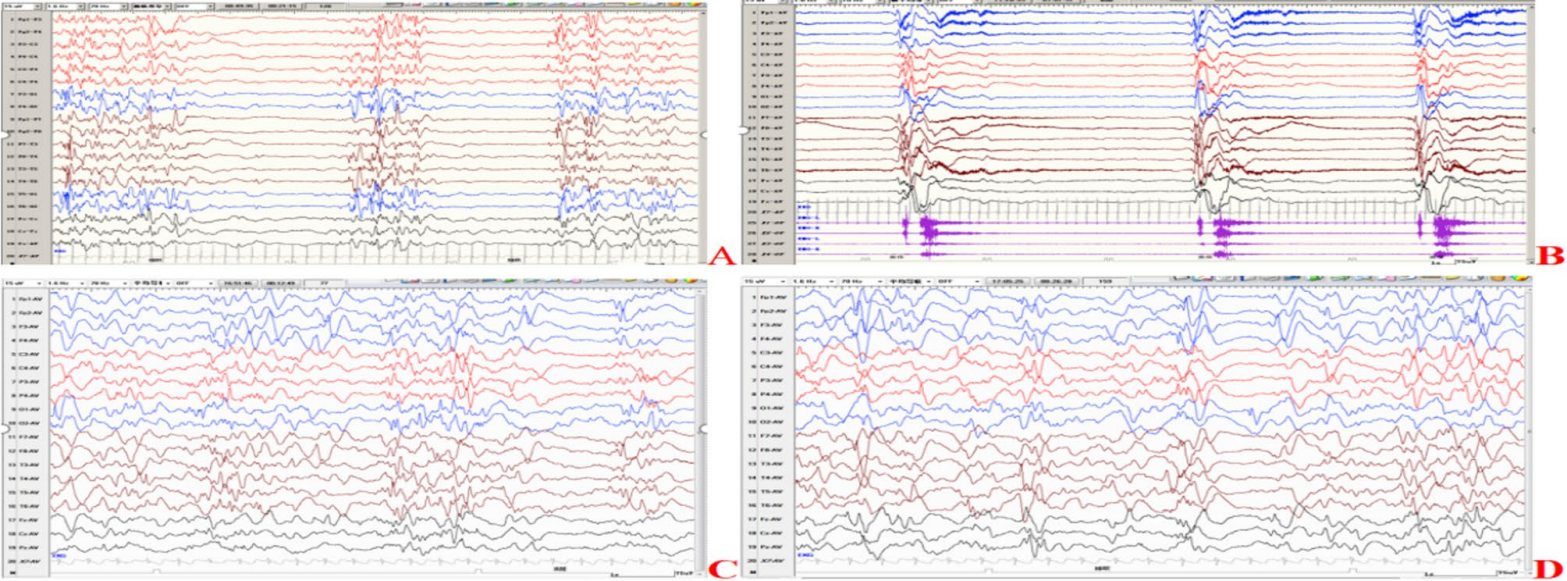
EEG of case 1 at one month of age (**A, B**) and case 2 at three months of age (**C, D**). A demonstrates interictal burst-suppression pattern; B shows ictal period which is epileptic spasms. C and D show interictal atypical hypsarrhythmia

Brain MRI revealed no significant abnormalities in cases 8 and 9, but bilateral frontotemporal subarachnoid widening in the remaining cases. Brain MRI showed delayed myelination of the white matter in cases 1, 2 and 6, which were all non-specific changes. EEG in cases 1–7 with epilepsy exhibited slowed background activity and abnormal discharges. Interictal EEG in cases 1–6 demonstrated multifocal or generalized epileptiform discharges. Additionally, a burst-suppression pattern was observed in cases 1 and 5 at age 2 months, while hypsarrhythmia occurred in cases 2, 3, 5, and 6 at age 3–4 months (Fig. 2). In contrast, case 7 showed focal epileptiform discharges in left mid-temporal region. Clinical seizures were monitored in cases 1, 2, 3, 5 and 6, including tonic seizures, epileptic spasms, and focal motor seizures. No epileptiform discharges were detected on EEG in cases 8 and 9, who lacked epileptic seizures. In addition, laboratory tests, including blood biochemistry, serum ammonia and lactate, urine and blood metabolic screenings were unremarkable in all cases. Echocardiograms and abdominal ultrasound examinations yielded normal results.

Regarding antiepileptic treatment, cases 1, 2 and 7 achieved seizure control after a combination of 2–3 ASMs, whereas cases 4, 5 and 6 exhibited refractory seizures despite trials of ≥ 4 oral ASMs. Case 3 did not respond to intravenous adrenocorticotropic hormone (ACTH) but responded to combined ACTH/ketogenic diet therapy. Conversely, case 6 was refractory to both ACTH and the ketogenic diet. For the efficacy of specific ASM, cases 1, 2 and 4 did not respond to phenobarbital, midazolam and levetiracetam, whereas topiramate demonstrated significant efficacy in these patients as well as in cases 3 and 6. At the last follow-up, cases 1, 2 and 7 were seizure-free for 0.5–2.5 years, cases 3 and 4 showed > 50% seizure reduction; and no significant reduction occurred in cases 5 and 6. Rechecked EEG revealed reduction or normalization of abnormal discharges in seizure-controlled/improved patients, with resolution of burst-suppression patterns (cases 1–2) and hypsarrhythmia (case 3).

During a follow-up period of 0.8–3.5 years, case 1 died at 1 year and 2 months of age due to recurrent infections and severe pneumonia despite seizure control. The remaining 8 cases showed varying degrees of mental and motor developmental delay. Cases 2–6 who are at 10 months to 3 years and 7 months age demonstrate severe neurodevelopmental impairment: Case 2 (seizure-controlled) achieved ambulation after age 2 years but with abnormal gait posture, while the other four cases 3–6 remain non-ambulatory and non-verbal without involuntary movements. Case 7 is twelve years old and has an IQ of 65 on the C-WISC (normal is 90–100), comorbid attention deficit hyperactivity disorder (ADHD) and choreoathetosis. Cases 8 and 9 are currently three and four years old respectively. They are still unable to walk and talk with marked involuntary movements and have a DQ below 50 which indicates severe developmental delay but have not present with seizures, although they are undergoing rehabilitation.

### Genetic variants analysis

Table 2 summarizes the *GNAO1* variants in the nine cases, all of which are *de novo* heterozygous missense variants. Of these, c.808 A > C (p.Asn270His), c.808 A > G (p.Asn270Asp), c.118G > C (p.Gly40Arg), c.521 A > G (p.Asp174Gly), c.687 C > A (p.Ser229Arg) and c.709G > A (p.Glu237Lys) have been reported in the Human Gene Mutation Database (HGMD) as known pathogenic variants [8–13]. Additionally, a different amino acid substitution c.119G > A (p.Gly40Glu) at the same position as c.119G > C (p.Gly40Ala) in case 1 has been reported [14], while c.17G > T (p.Ser6Ile) and c.748 C > T (p.Leu250Phe) are novel variants. All variants were classified as pathogenic or likely pathogenic according to ACMG guidelines.

**Fig. 3:**
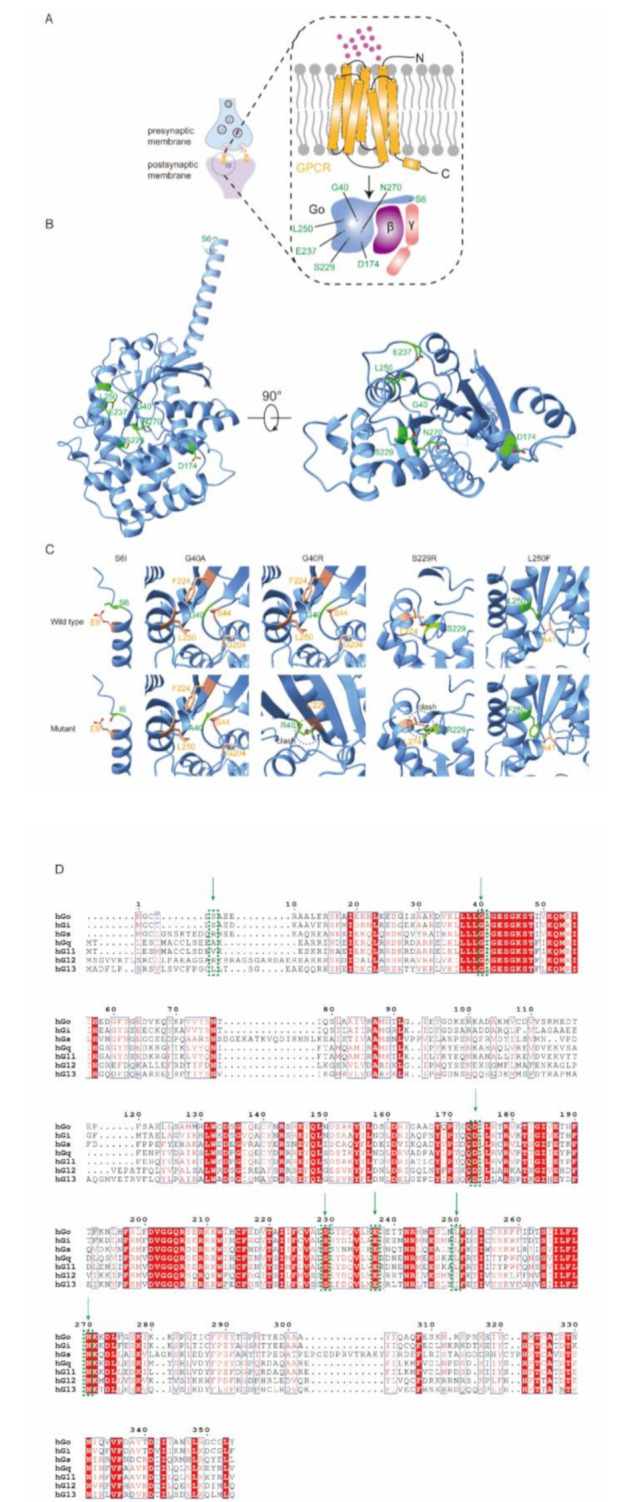
Impact of the variants on the protein. (**A**) Cartoon model of the heterotrimeric G-protein coupled-receptor in the synaptic cleft. (**B**) Location of the variants on the human Gαo subunit. The structure model is from the Alphafold2 database. Residues at the variant sites are depicted in green. Left panel: front view; right panel: bottom view. (**C**) Interactions between amino acids at the variant sites (green) and surrounding residues (orange) of p.S6I, p.G40A, p.G40R, p.S229R and p.L250F. Top panel: wild type; bottom panel: mutants. (**D**) Sequence alignment of human Gα subunits Go, Gi, Gs, Gq, G11, G12, and G13. Amino acids at the variant positions are circled with green dotted lines

**Fig. 4:**
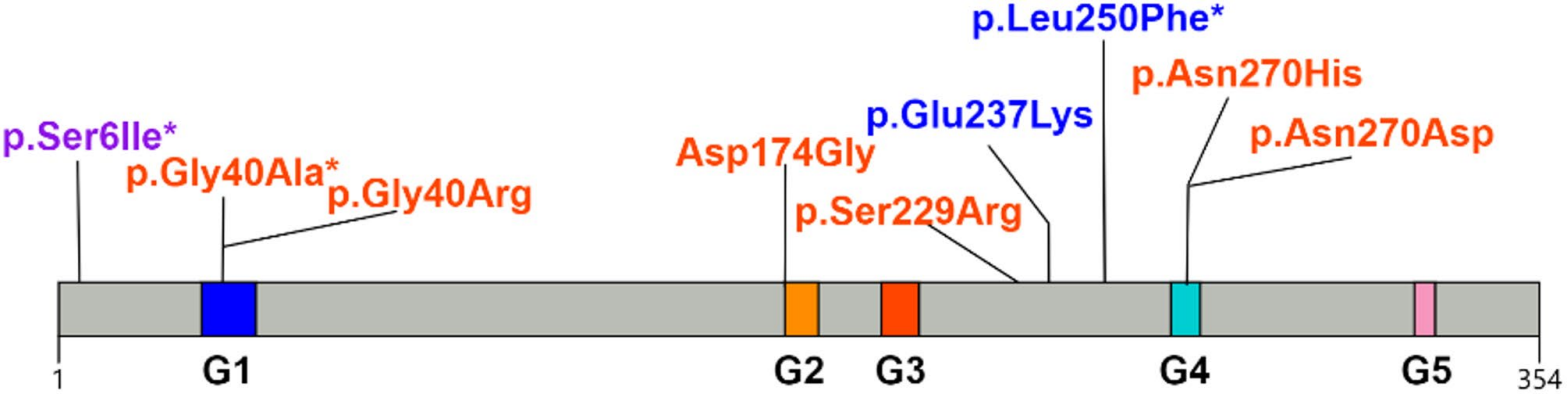
Schematic representation of the variant profile and protein structure of the GNAO1 gene in nine children Cases 1–6 are Developmental and Epileptic Encephalopathy 17 (DEE17; deep red mark): p.Gly40Ala*, p.Asn270His, p.Asn270Asp, p.Gly40Arg, p.Asp174Gly, p.Ser229Arg; the seventh case was of an intermediate type (purple marked): p.Ser6Ile*; cases 8–9 are Neurodevelopmental Disorder with Involuntary Movements (NEDIM: dark blue mark) p.Leu250Phe*, p.Glu237Lys. Previously unreported variants identified in this study are marked with *

Protein tertiary structure prediction was performed for three novel variants (cases 1, 7 and 8) and the reported c.687 C > A variant in case 6 who presented with early-onset infantile epileptic spasms, profound global developmental delay, and lack of response to multiple ASMs and ketogenic diet, and was thus considered to have the most severe clinical manifestation in this cohort (Fig. 3). The hydrogen bond of p.Gly40Ala remains unchanged, but the side-chain carbon atom interacts with the oxygen atom of cysteine at position 225. For p.Ser6Ile, the original serine at position 6 forms three hydrogen bonds with glutamic acid at position 9 and one hydrogen bond with arginine at position 10. The mutation to isoleucine results in the disappearance of the two hydrogen bonds of glutamic acid at position 9. p.Leu250Phe preserves the hydrophobic interaction with Ala41, whereas p.Ser229Arg identified in the most severe phenotype case (case 6) induces the mutation of serine to arginine at position 229 creates a steric clash with leucine at position 274, which is not present in the wild-type Gα protein and leads to a local structural change. Specific distribution map of protein domain sites of GNAO 1 in 9 children is showed in Fig. 4 (Fig. 4).

## Discussion

*GNAO1* encephalopathy has a broad phenotypic spectrum, the most common symptom being seizure. Seizure types are mainly generalized tonic-clonic seizures, myoclonic seizures, epileptic spasms, and focal seizures. The EEG typically shows multifocal or generalized spike-wave and polyspike-wave discharges, some of which presenting with burst-suppression pattern or hypsarrhythmia [2, 3]. In the present study, the electro-clinical phenotype of cases 1 and 5 could be further specifically diagnosed as Ohtahara syndrome and cases 2, 3 and 6 as infantile epileptic spasm syndrome, which is consistent with the typical electro-clinical phenotype of DEE17 previously reported [3, 10]. Notably, we performed genetic testing on nine cases and identified three novel *GNAO1* variants. Our study expanded the genotype-phenotype spectrum of GNAO1-related brain disorders.

The *GNAO1*-related phenotypes reported to date is expanding. In this cohort, cases 1–6 exhibited typical DEE, while cases 8 and 9 manifested NEDIM. Case 7 has a late onset of epilepsy, with focal seizures and focal EEG epileptiform discharges. The seizures in case 7 are easily controlled by ASM with an earlier and more prominent appearance of involuntary movement, thus we consider that this case may be an intermediate phenotype between DEE17 and NEDIM. In addition, Wirth et al. recently reported a mild phenotype in a group of 24 patients presenting with nonprogressive generalized or focal/segmental upper-body dystonia appearing beyond infancy, associated with dysarthria, but without epilepsy or developmental delay [15]. In conclusion, there is significant overlap in clinical symptoms in the majority of patients, with *GNAO1*-related neurodevelopmental disorders comprising a continuous spectrum rather than distinct entities. In the future, prospective longitudinal clinical investigations are needed to document the individual disease progression.

Current genomic landscapes (Human Gene Mutation Database: 89 *GNAO1* variants, 65 missense, as of February 2023) confirm missense substitutions as the primary pathogenic subtype, consistent with our nine de novo cases [5, 10, 14]. While functional consequences show correlation with structural localization–specifically GTP-binding domain perturbations where Gly40 variants associate with severe phenotypes, C-terminal variants with mild manifestations, and residues 207–221 with isolated dyskinesia/hypotonia per McKenna et al. [14]. In the present study, all variants are located in the GTP-binding region except p.Ser6Ile and p.Asp174Gly. Phenotypes of cases 2–6 and 9 align with prior reports for their respective variants [8–13] with p.Ser6Ile’s N-terminal location possibly explaining case 7’s intermediate phenotype. However, significant exceptions challenge deterministic models: variants affecting the amino acids of the entire protein can cause epilepsy [14]; Wirth et al. reported milder phenotypes with variants proximal to established hotspots [15], and presumed loss-of-function variants (nonsense/gene deletion) cause late-onset dystonia sans ID. Feng et al. concluded that seizures in patients with *GNAO1* variants are associated with reduced or loss of G protein expression, whereas patients with movement disorder but no seizure may have variants resulted in elevated or insignificant G protein expression [6, 8]. Paradoxically, Muntean et al.‘s findings demonstrated that P-loop variants (Gly42Arg/Ser47Gly/Ile56Thr) disrupt Gβγ binding, while switch-region variants (Gly203Arg/Arg209Cys/Glu246Lys) exert dominant-negative effects through wild-type Gαo inhibition—collectively indicating combined LOF/dominant-negative pathomechanisms [16]. In our study, structural predictions further demonstrate novel variants (cases 1, 7, 8) and case 6’s variant induce conformational changes in the conserved GTP-domain (Fig. 3). These variants may have an effect on the protein structure and function, but the exact effect is difficult to estimate. The phenotype-genotype correlations of *GNAO1*-related disorder are quite nuanced, and the exact mechanism of individual pathogenic variant needs to be considered on a case-by-case basis.

Regarding treatment, good anti-seizure efficacy of topiramate is observed in the present study. *GNAO1* variants lead to reduced inhibition of its mediated Ca^2+^ currents resulting in seizures or dyskinesia [17]. Topiramate has been reported to reduce seizures and also improve dyskinesia in one study, which may be related to the upregulation of inhibition of Ca^2+^ voltage channel activation [18]. In this cohort, the symptoms were alleviated in five out of six cases after the treatment of topiramate, and one of them was maintained seizure-free on monotherapy. Our results demonstrated that topiramate can be used as an effective ASM for clinical treatment of epilepsy and DEE17.

**Table 1:** Clinical manifestations of the nine patients

	Sex	Age at diagnosis	Age of onset	Psychomotor development at diagnosis	Epilepsy			Dystonia	Involuntary movements		Prognosis		
					Seizure types	Interictal EEG	Antiseizure treatment				Epilepsy		Development
1	Male	2 months	25 days	Unable to hold head upright, poor tracking of vision	Tonic-clonic seizure, myoclonic seizure	Generalized or multifocal spikes and spike-waves, with burst-suppression	PB, MDZ, LEV, TPM	Hypotonia	-		Seizure controlled for 0.5 year	Died due to infection at 1year and 2 months of age	
2	Male	4 months	2.5 months	Unstable upright head hold, poor tracking vision and hearing, could not be made to laugh	Epileptic spasms	Multifocal spikes, sharp waves, and spike-waves, with hypsarrhythmia	PB, LEV, ACTH, *TPM*	Hypotonia	-		Seizure controlled for 2.2 years		Able to walk alone but unsteady gait, speaks only a few words
3	Female	4 months	11 days	Unstable upright head hold, poor tracking vision and hearing, could not be made to laugh	Tonic-clonic seizure, myoclonic seizure, epileptic spasms	Generalized spikes, sharp waves, and spike-waves, with hypsarrhythmia	ACTH, *TPM*, *VPA*, *CLB*, *KDT*	Hypotonia	-		Seizure reduction > 50%		Could not sit, stand and walk, nonverbal
4	Female	1 month	2 days	Poor tracking vision and hearing, no spontaneous laugh	Tonic-clonic seizure, myoclonic seizure	Multifocal spikes, sharp waves, and spike-waves predominantly during sleep	PB, MDZ, *LEV*, *TPM*	Hypotonia	-		Seizure reduction > 50%		Could not sit, stand and walk, nonverbal
5	Female	4 months	1.7 months	Unstable upright head hold, poor tracking vision and hearing, could not be made to laugh	Focal tonic seizure, myoclonic seizure, epileptic spasms	Burst-suppression at 2 months, hypsarrhythmia at 5 months	OXC, *VPA*, *TPM*, *CZP*	Hypertonia	-		No reduction in seizure frequency		Could not sit, stand and walk, nonverbal
6	Female	3 months	12 days	Poor tracking vision and hearing, no spontaneous laugh	Focal motor seizure, epileptic spasms, tonic seizure	Generalized or multifocal spikes, sharp waves, and spike-waves, with hypsarrhythmia	OXC, ACTH, CLB, *TPM*, PB, *VPA*, *VGB*, KDT	Hypotonia	-		No reduction in seizure frequency		Could not sit, stand and walk, nonverbal
7	Male	8 years	2 years	Able to walk alone but unsteady gait, speaks only a few words	Focal seizures and focal to bilateral tonic-clonic seizures	Slow waves and spike-waves predominantly in left temporal region	*LEV*, OXC, *VPA*	Normal	Choreoathetosis		Seizure controlled for 2.5 years		Able to walk alone but unsteady gait, speak simple phrases but little active language expressions, attention deficit
8	Female	2.5 years	4 months	Could not sit, stand and walk, nonverbal	-	-	-	Hypertonia	Choreoathetosis	-			Could not sit, stand and walk, nonverbal
9	Female	4 years	3.5 months	Could not sit, stand and walk, nonverbal	--	-	-	Hypotonia	Choreoathetosis	-			Could not sit, stand and walk, nonverbal

Abbreviations: EEG, electroencephalography; PB, phenobarbital; MDZ, midazolam (intravenous); LEV, levetiracetam; TPM, topiramate; ACTH, adrenocorticotropic hormone (intravenous); VPA, valproate acid; OXC, oxcarbazepine; CLB, clobazam; CZP, clonazepam; VGB, vigabatrin; KDT, ketogenic diet therapy. Note: underlining indicates current treatment

**Table 2:** The *GNAO1* gene variants in the nine cases

	1	2	3	4	5	6	7	8	9
Diagnosis	DEE17	DEE17	DEE17	DEE17	DEE17	DEE17	Intermediate phenotype	NEDIM	NEDIM
Exon	2	7	7	2	5	6	1	7	6
Nucleotide changes	c.119G > C	c.808 A > C	c.808 A > G	c.118G > C	c.521 A > G	c.687 C > A	c.17G > T	c.748 C > T	c.709G > A
Amino acid change	p.Gly40Ala	p.Asn270His	p.Asn270Asp	p.Gly40Arg	p.Asp174Gly	p.Ser229Arg	p.Ser6Ile	p.Leu250Phe	p.Glu237Lys
Reported in HGMD	No (c.119G > A p.Gly40Glu has been reported)	Yes	Yes	Yes	Yes	Yes	No	No	Yes
gnomAD AF	0	0	0	0	0	0	0	0	0
Evidence for pathogenicity based on ACMG	PS2 + PM1 + PM2 + PP2 + PP3	PS1 + PS2 + PM1 + PM2 + PP2 + PP3	PS2 + PM1 + PM2 + PM5 + PP2 + PP3	PS1 + PS2 + PM1 + PM2 + PP2 + PP3	PS2 + PS4_Moderate + PM2_Supporting + PP2 + PP3	PM6 + PM2-supporting + PP2 + PP3	PS2 + PM1 + PM2 + PP3	PS2 + PM1 + PM2 + PP2 + PP3	PS2 + PS4 + PM1 + PM2 + PP2 + PP3 + PP4
Interpreted pathogenity	Pathogenic	Pathogenic	Pathogenic	Pathogenic	Likely pathogenic	Likely pathogenic	Likely pathogenic	Likely pathogenic	Pathogenic

Abbreviations: DEE17, developmental and epileptic encephalopathy 17; NEDIM, neurodevelopmental disorder with involuntary movements; HGMD, human gene mutation database; AF, allele frequency

## Conclusions

In conclusion, we analyzed the clinical characteristics and genetic variants of a cohort of nine Chinese children with *GNAO1* variants and highlight the correlation of genotypes and phenotypes. The study showed that *GNAO1* variants resulted in a group of broad phenotypic spectrums, with the majority of patients having psychomotor developmental delay, in which DEE17 patients typically have refractory epilepsy with early onset of multiple seizure types, while NEDIM is characterized by involuntary movements and dystonia. Notably, we identified three novel pathogenic/likely pathogenic missense variants: p.Ser6Ile, p.Gly40Ala and p.Leu250Phe.Three novel variants and case 6 with the most severe phenotype resulted in varying degrees of local structural chang. These findings highlight the complexity of genotype-phenotype correlations, and the location of the variant within the protein may have some relevance to the phenotype. Further research and collection of more cases are needed in the future, and this ongoing international collaborative research.

## Supplementary Information

Below is the link to the electronic supplementary material.

Supplementary Material 1

## Data Availability

All data generated or analyzed for the study are available from the corresponding author upon reasonable request.

## Abbreviations

DEE 17: Developmental and epileptic encephalopathy 17
NEDIM: Neurodevelopmental disorder with involuntary movements
ACMG: American College of Medical Genetics and Genomics
MRI: Magnetic resonance imaging
HGMD: Human Gene Mutation Database

## References

[1] Briere, L., Et,al. (2023). GNAO1-Related Disorder. In M. P. Adam (Eds.) et. al., GeneReviews®. University of Washington, Seattle.Copyright © 1993-2025, University of Washington, Seattle. GeneReviews is a registered trademark of the University of Washington, Seattle. All rights reserved.

[2] Nakamura K, et al. De Novo mutations in GNAO1, encoding a Gαo subunit of heterotrimeric G proteins, cause epileptic encephalopathy. Am J Hum Genet. 2013;93(3):496–505. 23993195 10.1016/j.ajhg.2013.07.014 PMC3769919

[3] Saitsu H, et al. Phenotypic spectrum of GNAO1 variants: epileptic encephalopathy to involuntary movements with severe developmental delay. Eur J Hum Genet. 2016;24(1):129–34. 25966631 10.1038/ejhg.2015.92 PMC4795232

[4] Kulkarni N, et al. Progressive movement disorder in brothers carrying a GNAO1 mutation responsive to deep brain stimulation. J Child Neurol. 2016;31(2):211–4. 26060304 10.1177/0883073815587945

[5] Danti FR, et al. GNAO1 encephalopathy: broadening the phenotype and evaluating treatment and outcome. Neurol Genet. 2017;3(2):e143. 28357411 10.1212/NXG.0000000000000143 PMC5362187

[6] Feng H, et al. A mechanistic review on GNAO1-associated movement disorder. Neurobiol Dis. 2018;116:131–41. 29758257 10.1016/j.nbd.2018.05.005

[7] Richards S, et al. Standards and guidelines for the interpretation of sequence variants: a joint consensus recommendation of the American college of medical genetics and genomics and the association for molecular pathology. Genet Med. 2015;17(5):405–24. 25741868 10.1038/gim.2015.30 PMC4544753

[8] Huijie F et al. Movement disorder in GNAO1 encephalopathy associated with gain-of-function mutations. Neurology, 2017. 89(8). 10.1212/WNL.0000000000004262 PMC5580866 28747448

[9] Atsushi T et al. Comprehensive analysis of coding variants highlights genetic complexity in developmental and epileptic encephalopathy. Nat Commun, 2019. 10(1). 10.1038/s41467-019-10482-9 PMC6555845 31175295

[10] Xiaoling Y et al. Phenotypes of GNAO1 variants in a Chinese cohort. Front Neurol, 2021. 12(0). 10.3389/fneur.2021.662162 PMC8193119 34122306

[11] Weihao L et al. Treating GNAO1 mutation-related severe movement disorders with oxcarbazepine: a case report. Transl Pediatr, 2022. 11(9). 10.21037/tp-22-297 PMC9561508 36247896

[12] Tommaso S et al. Phenomenology and clinical course of movement disorder in GNAO1 variants: results from an analytical review. Parkinsonism Relat Disord, 2019. 61(0). 10.1016/j.parkreldis.2018.11.019 30642806

[13] De novo mutations. In synaptic transmission genes including DNM1 cause epileptic encephalopathies. Am J Hum Genet, 2014. 95(4). 10.1016/j.ajhg.2014.08.013 PMC4185114 25262651

[14] McKenna K et al. Spectrum of neurodevelopmental disease associated with the GNAO1 Guanosine triphosphate-binding region. Epilepsia, 2019. 60(3). 10.1111/epi.14653 PMC6452443 30682224

[15] Thomas W et al. Highlighting the dystonic phenotype related to GNAO1. Mov Disord, 2022. 37(7). 10.1002/mds.29074 PMC9545634 35722775

[16] Brian S. M., Gαo is a major determinant of cAMP signaling in the pathophysiology of movement disorders. Cell Rep, 2021. 34(5). 10.1016/j.celrep.2021.108718 PMC7903328 33535037

[17] Jason M. K., Gain-of-function mutation in Gnao1: a murine model of epileptiform encephalopathy (EIEE17)? Mamm Genome, 2014. 25(0). 10.1007/s00335-014-9509-z PMC4042023 24700286

[18] Saori S et al. A case of severe movement disorder with GNAO1 mutation responsive to topiramate. Brain Dev, 2016. 39(5). 10.1016/j.braindev.2016.11.009 27916449

