# Supplementary material for: Identification of three novel GNAO1 variants in a Chinese cohort with GNAO1 encephalopathy: expanding the clinical and genetic spectrum
Source: Orphanet J Rare Dis. 2025 Aug 18;20:440. doi: 10.1186/s13023-025-03984-x (PMC12363038; doi:10.1186/s13023-025-03984-x)
Supplement: Supplementary file 1 — Supplementary Material 1 [file 13023_2025_3984_MOESM1_ESM.docx]

**Response letter**

Dear Editors and Reviewers:

Thank you for your letter and for the reviewers’ comments concerning our manuscript entitled “Clinical phenotype and genotype analysis of GNAO1 encephalopathy: a cohort of Chinese children” (OJRD-D-25-00243). Those comments are all valuable and helpful for revising and improving our paper, as well as the important guiding significance to our researches. We have studied comments carefully and have made correction which we hope meet with approval. Revised portion are marked in red in the paper. The main corrections in the paper and responses to the reviewers’ comments are as follows:

Reviewer #1: This is a very useful work because it adds three previously unpublished variants to the literature. I am not sure why the authors do not indicate this in their title, as many more people are likely to read an article indicating the finding of 3 new pathogenic variants, rather than what at first glance seems to be just a review of a cohort of patients with a known disease. The stated objective of this paper is "to summarize." With three new variants that are pathogenic/likely pathogenic, this work is more than a summary. If these are truly previously-unpublished variants, I suggest the authors use a more pointed title such as, for example: "Identification of three new gene variants in GNAO1 encephalopathy, and their placement in the clinical spectrum of a cohort of nine patients", or some equivalent title that more accurately reflects the significance of the paper.

Response: Thank you for your helpful comments. We have revised the title to: “**Ident-ification of three novel GNAO1 variants in a Chinese cohort with GNAO1 encephalopathy: expanding the clinical and genetic spectrum”**.
The conclusion should be rewritten accordingly to reflect this increased emphasis on the new variants as the most important aspect of the work.

Response: We agree with your suggestion about conclusion and we have rewritten it to reflect this emphasis (page 9).
Along the same lines, I suggest highlighting the new variants with an asterisk in figure 4, with an indication in the figure legend.

Response: Thank you for your reminding. We have highlighten the new variants with asterisks in figure 4 and indicated it in figure legend.

Please provide a specific credit for the cartoon in figure 3A.

Response: Thank you for your reminding, the cartoon in figure 3A was made by Adobe Illustrator software. We have added it in figure legend.
The data availability statement is incomplete.

Response: Thank you for your suggestion. We have revised this statement (page 10).
There are errors of English language usage and typographical errors scattered throughout the text, too many to enumerate. They do not impede understanding of the work, but it would improve the paper if they were corrected.

Response: Thank you for referring to the errors. We have thoroughly edited the manuscript for grammar and clarity (highlighted in red).

Reviewer #2: The manuscript is very interesting and original.

I suggest that manuscript should be rewritten to be emphasized (in introduction and discussion) its main value: the case series revealed the two relevant phenotypes of GNAO1 encephalopathy (DEE 17 and NEDIM) in a distinct population (Chinese) not previously reported in literature.

Response: Thank you for your suggestion. We have rewritten the introduction and discussion, please see page 3 and page 7-9.

I suggest that more populacional data should be included in the text of manuscript like prevalence of disorder, historical of report of the two phenotypes, underdiagnosis estimate and prevalence in specific populations.

Response: Thank you for your comments. We have added it in introduction, please see page 3, line 12.

I suggest that discussion should be rewritten because there is a lot of paragraphs trying to point a genotype-phenotype correlation in *GNAO1* encephalopathy and the authors agree that this correlation is not possible at the end. Other features about the data obtained and important results of manuscript could be considered in discussion like the efficacy of some anticonvulsant drugs.

Response: We appreciate your friendly comments. We have rewritten discussion, please see page 8.

Regarding that clinical characterization of a case series is the main objective, other clinical features should be mentioned in results as gastrointestinal problems, sleep disorders, vision/hearing impairments, and some data about movement disorder treatment of patients 7, 8 and 9.

Response: Thank you for your suggestion. The main phenotypes caused by GNAO1 gene mutations are developmental epileptic encephalopathy type 17 and involuntary movements. During the subsequent visits and follow-up, the child's feeding difficulties, sleep disorders, visual and hearing impairments were not obvious, and the parents did not mention them. Example 7-9 showed improvement in involuntary movements and dystonia through rehabilitation functional training and symptomatic treatment.

I suggest several editions that are detailed in the following topics.

Response: Thank you for your helpful comments. We have revised these topics according to your suggestion.

1) In the manuscript abstract: avoid to use de the term 'case' and the term 'children were collected'.

Response: Thank you for your recommendation. We have replaced these words and marked in red.

2) In the abstract, elaborate a conclusion more related to the results.

Response: Thank you for your suggestion. We have rewritten the conclusion in the abstract. Please see page 3, line 20-23.

3) In the abstract, 'All' or 'All patients'.

Response: Thank you for your comments. We have revised these parts and marked in red.

4) In the abstract, try do not repeat the word 'with' so many times

Response: Thank you for your correction. We have revised it.

5) In all manuscript, use italics for 'de novo'.

Response: Thank you for your correction. We have turned “de novo” into “*de novo*” and marked in red.

6) The first paragraph of introduction should not be about the protein structure or GNAO1 function, but it should be about what is known about the disorder.

Response: We thank you and agree with your opinions. We have rewritten the introduction.

7) In material and methods, there is an unnecessary repetition regarding the statement of obtaining the consent form (1st and 3rd paragraphs).

Response: Thank you for your reminding. We have deleted the repeat part in 3rd paragraph in material and methods.

8) It should be mentioned how many patients were followed by telephone.

Response: Thank you for your reminding. All 9 children patients were followed up regularly by phone or in person at the outpatient departmen.

9) I suggest to use 'Brain MRI' instead of 'Cranial MRI'.

Response: Thank you for your suggestion. We have replaced all instances of 'Cranial MRI' with 'Brain MRI' throughout the manuscript.

10) In Results: page 7, line 6, check the correct use of 'who'; line 17, check the use of 'while'.

Response: Thank you for correcting it. We have revised this error. Please see page 7, lines 6 -7.

11) I suggest to also report the EEG results of patient 8 and patient 9.

Response: Thank you for your comments. We have added the EEG results of case 8 and case 9 in page 5, line 22-24.

12) In Results, it is not clear what the definition of effective treatment for seizures is.

Response: We thank for your comments. We have added detailed description about the effects of ASM treatment. Please see page 4, line 12-18.

13) Standardize how to write numerals (in full or symbols) throughout the manuscript text.

Response: Thank you for your reminding. We have standardized numerals in the manuscript and marked in red.

14) The description of variants should follow HGVS Nomenclature for protein sequence variants. Avoid to use Letters for aminoacids.

Response: Thank you for your correction. We have revised the description of vairants according to the HGVS Nomencature and marked in red.

15) Why patient 6 was considered to present 'the most severe clinical manifestation in this cohort'?

Response: Thank you for your comments. We have referred the reason and marked it in red in the manuscript, please see page 7, line 3-6.

16) In discussion, page 10, line 9, I suggest do not use the conjunction 'but' or change it; line 14, the sentence is missing the verb.

Response: Thank you for correcting it. We have revised the error and marked in the manuscript. Please see page 8, line 1.

17) In discussion, page 10, the last sentence of the 1st paragraph is out of context and it makes no sense to mention only these two genes for the differential diagnosis of GNAO1 encephalopathy.

Response: Thank you for your recommendation. We have deleted the sentence.

18) In discussion, it should be emphasized that review of this Chinese cohort reinforces that phenotype-genotype correlation in GNAO1 encephalopathy is nuanced.

Response: Thank you for your helpful comments. We have rewritten this part, please see page 8-9.

19) More emphasis should be done in the manuscript for the role of topiramate in treatment of seizures based in results of cohort.

Response: We appreciate your useful comments. We have added the role of topiramate in treatment of seizures in discussion, please see page 9.

20) I suggest remove the last sentence of discussion because it is out of context.

Response: Thank you for your recommendation. We have deleted the sentence.

21) I suggest to include this following reference in the manuscript: Briere L, Thiel M, Sweetser DA, et al. GNAO1-Related Disorder. 2023 Nov 9. In: Adam MP, Feldman J, Mirzaa GM, et al., editors. GeneReviews®

Response: Thank you for your comments. We have referenced the literature in the manuscript. Please see reference 1.
